# Ebola Virus Antibodies in Fruit Bats, Ghana, West Africa

**DOI:** 10.3201/eid1807.111654

**Published:** 2012-07

**Authors:** David T.S. Hayman, Meng Yu, Gary Crameri, Lin-Fa Wang, Richard Suu-Ire, James L.N. Wood, Andrew A. Cunningham

**Affiliations:** University of Cambridge, Cambridge, UK (D.T.S. Hayman, J.L.N. Wood);; Zoological Society of London, London, UK (D.T.S. Hayman, A.A. Cunningham);; Animal Health and Veterinary Laboratories Agency, Weybridge, UK (D.T.S. Hayman);; Colorado State University, Fort Collins, CO, USA (D.T.S. Hayman);; CSIRO Livestock Industries, Geelong, Victoria, Australia (M. Yu, G. Crameri, L.-F. Wang);; and Ghana Forestry Commission, Accra, Ghana (R. Suu-Ire)

**Keywords:** viruses, Ebola virus, Ebolavirus sp., Reston Ebola virus, REBOV, Zaire Ebola virus, ZEBOV, African fruit bats, Africa, Epomops franqueti, Epomophorus gambianus, Hypsignathus monstrosus, Nanonycteris veldkampii, Eidolon helvum, viruses, Ghana

**To the Editor:** Fruit bats are the presumptive reservoir hosts of Ebola viruses (EBOVs) (genus *Ebolavirus*, family *Filoviridae*). When transmitted to humans and nonhuman primates, EBOVs can cause hemorrhagic fevers with high case-fatality rates. In 2008, we detected Zaire EBOV (ZEBOV) antibodies in a single migratory fruit bat (*Eidolon helvum*) from a roost in Accra, Ghana (*1*). This bat is common in sub-Saharan Africa and lives in large colonies, often in cities. The flight of an individual *E. helvum* bat during migration has been recorded as >2,500 km (*2*).

To understand whether the single seropositive *Eidolon helvum* bat was evidence of EBOV circulation in the Greater Accra Region or elsewhere in sub-Saharan Africa, we tested serum of 88 nonmigratory fruit bats from the surrounding region of Ghana. Serum samples had been collected, as previously described (*3**,**4*), during May–June 2007 from fruit bats in woodland and tropical forest habitats in southern Ghana within 180 km of Accra. Initial screening for EBOV antibodies was conducted by using ELISA with a 1:1 mixture of recombinant nucleoprotein (NP) of ZEBOV and Reston EBOV (REBOV). Proteins were expressed in an *Escherichia coli* expression vector with a polyhistadine tag (*1**,**5*). Samples with optical density (OD) readings 3-fold above the mean OD of 2 negative control serum samples were considered EBOV-positive by ELISA. ELISA-positive samples were tested separately (at a dilution of 1:50) for reactivity against ZEBOV and REBOV NPs by using ELISA and Western blot (WB) as described (*1*). Each sample with positive results from both assays was retested at increasing dilutions to determine the highest dilution (endpoint titer) at which it remained positive (>3-fold above the OD for EBOV-negative sera).

We detected EBOV antibodies (1:1 mixture of both NP antigens, OD>0.7) in serum samples from 32 of 88 bats (10/27 *Epomops franqueti*, 14/37 *Epomophorus gambianus*, 7/16 *Hypsignathus monstrosus,* 1/4 *Nanonycteris veldkampii,* and 0/1 *Epomops buettikoferi*). When tested against an individual NP, 13 of the 32 EBOV-positive serum samples were positive for EBOV (OD >0.50). Of those 13 EBOV-positive samples, 9 were ZEBOV-positive only (from 3 *E. franqueti*, 4 *E. gambianus,* and 2 *H. monstrosus* bats), 3 were REBOV-positive only (from 2 *E. gambianus* and 1 *H. monstrosus* bats), and 1 sample from an *E. gambianus* bat was positive for both ZEBOV and REBOV. Seven samples that the EBOV NP ELISA identified as positive were also positive by WB (Table). Each WB-positive serum sample was positive for the EBOV antigen that it had been most reactive against in the ELISA: 5 WB test results were positive for ZEBOV (2 of those samples also bound to REBOV), and 2 bound to REBOV only. Serum samples with positive OD values at endpoint dilutions >1:50 were definitively positive by WB; whereas those with positive OD values at and endpoint dilution of 1:50 only could be positive, negative, or equivocal by WB (Table).

**Table:** ZEBOV- and REBOV-specific results of ELISA and Western blot analysis of serum from Ebola virus–positive fruit bats, Ghana*

Location, date, fruit bat species	Sex	Age	ELISA OD (endpoint titer dilution)			Western blot†	
			REBOV	ZEBOV		REBOV	ZEBOV
Sagyimase							
May 29, 2007							
* Epomops franqueti*	F	A	0.11 (1:50)	0.53 (1:200)		−	+
* E. franqueti*	F	SI	0.08 (1:50)	0.62 (1:100)		+	+
* Hypsignathus monstrosus*	F	A	0.12 (1:50)	1.08 (1:200)		+	+
* H. monstrosus*	F	SI	1.11 (1:200)	0.17 (1:50)		+	−
* H. monstrosus*	F	A	0.15	0.71		−	−
* H. monstrosus‡*	M	SI	0.05	0.11		−	−
May 30, 2007							
* E. franqueti*	F	A	0.1§	0.53§		−	+
Adoagyiri							
May 31, 2007							
* Epomophorus gambianus*	M	A	0.59	0.67		−	−
*E. franqueti*§	F	A	0.05	0.16		−	−
* E. gambianus*	F	A	0.19	0.55		−	−
* E. gambianus*	F	A	0.13 (1:50)	1.24 (1:800)		−	+
* E. gambianus*	F	SI	0.65 (1:200)	0.36 (1:50)		+	−
Oyibi							
June 2, 2007							
* E. gambianus*	M	A	0.51	0.63		−	−
Negative control (RAB691/d0)			0.19	0.27			
Positive control (RAB691/EBOV-N)			1.39	1.42		+	+

*ZEBOV, Zaire Ebola virus; REBOV, Reston Ebola virus; OD, optical density; A, adult; SI, sexually immature. †Only serum samples with ZEBOV- or REBOV- positive ELISA results (optical density >0.50) were tested. ‡Negative-control field serum samples. §Additional serum was not available for endpoint titer dilution determination.

Previous serum and viral antigen tests indicated the presence of EBOV among 2 of these bat species (*E. franqueti* and *H. monstrosus*) in Gabon, located in central Africa (*6*). Two others (*E. gambianus* and *N. veldkampii*) were not previously identified as potential reservoirs. Because these are nonmigratory fruit bats, our findings demonstrate that at least 1 serotype of EBOV circulates in bats in the Upper Guinean forest ecosystem in West Africa. These data might provide evidence that Taï Forest EBOV (TEBOV), formerly known as Côte d’Ivoire EBOV, circulates in this ecosystem among bats native to West Africa (*7*). EBOV antibody titers are highly correlated (*8*), but using TEBOV antigen might increase seroprevalence if TEBOV is the circulating virus. However, geographic location does not necessarily determine EBOV genetic relationships (*9*), and lack of cross-reactivity between serum samples positive for REBOV and ZEBOV in our study might indicate that divergent viruses circulate regionally, given phylogenetic and antigenic relationships between EBOV species (*7**–**10*).

We detected a relatively high proportion of EBOV-seropositive fruit bats in a relatively small sample size of mixed species. We suggest that the prevalence of EBOV in these tested bat species is greater than that previously detected in *E. helvum* bats (1/262 serum samples) (*1*). The higher estimated prevalence in these species occurred despite the fact that *E. helvum* bats live in large colonies comprising several million animals, which make the species an ideal host for acute RNA virus infections. The relatively low seroprevalence of EBOV among *E. helvum* bats compared with that among sympatric species is contrary to our findings for a lyssavirus and an uncharacterized henipavirus (*3**,**4*). Our results, therefore, lead us to question what factors (e.g., host, ecologic) limit EBOV circulation in straw-colored fruit bats. Virus isolation is required to characterize EBOVs circulating among fruit bats in Ghana, and additional testing, including longitudinal sampling of bats, is required to further investigate the epidemiology of EBOV in West Africa. Possible public health threats should also be investigated and addressed. These initial findings, however, suggest that the risk for human infection with EBOV might be greater from bat-human contact in rural and forest settings than from urban-roosting *E. helvum* bats.
